# High-resolution X-ray structure of Gln143Asn manganese superoxide dismutase captures multiple hydrogen peroxide-binding sites

**DOI:** 10.1107/S2053230X25009045

**Published:** 2025-10-23

**Authors:** Medhanjali Dasgupta, Katelyn Slobodnik, Erika A. Cone, Jahaun Azadmanesh, Thomas Kroll, Gloria E. O. Borgstahl

**Affiliations:** a Eppley Institute for Research in Cancer and Allied Disease University of Nebraska Medical Center Omaha NE68198 USA; b Stanford Synchrotron Radiation Lightsource SLAC National Accelerator Laboratory Menlo Park CA94025 USA; The Scripps Research Institute, USA

**Keywords:** manganese superoxide dismutases, peroxide binding, metalloenzymes, X-ray crystallography

## Abstract

Human mitochondrial manganese superoxide dismutase (MnSOD) converts superoxide into hydrogen peroxide and molecular oxygen, serving as a key defence against oxidative damage. Despite extensive studies, the full structural characterization of H_2_O_2_-binding sites in MnSOD remains largely unexplored. Previous H_2_O_2_-soaked MnSOD structures have identified two distinct H_2_O_2_-binding sites: one directly ligated to the catalytic manganese (LIG position) and another at the active-site gateway (PEO position) between the second-shell residues Tyr34 and His30. In this study, a kinetically impaired Gln143Asn MnSOD variant is used to trap and explore additional H_2_O_2_-binding sites beyond the second-shell solvent gate. In the wild-type enzyme, Gln143 mediates proton transfers with the manganese-bound solvent (WAT1) to drive redox cycling of the metal, which is necessary for effective superoxide dismutation. Substitution with Asn stalls catalysis because the increased distance from WAT1 disrupts critical proton-coupled electron-transfer (PCET) events, and the redox cycling of the active-site metal is impaired. This, in turn, stalls the electrostatic cycling of positive charge on the enzyme surface and enhances the likelihood of trapping transient H_2_O_2_-bound states in this variant. The results reveal several H_2_O_2_ molecules leading up to the active site, in addition to the canonical LIG and PEO positions.

## Abstract 2

A high-resolution X-ray structure of a Gln143Asn variant of manganese superoxide dismutase (MnSOD) reveals multiple hydrogen peroxide-binding sites beyond the canonical LIG and PEO binding positions within the active site. These findings expand the known landscape of product peroxide-bound states in MnSOD.

## 1. Introduction

Human manganese superoxide dismutase (MnSOD) is a mitochondrial matrix metalloenzyme that catalyzes the dismutation of reactive superoxide radicals (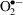), generated as byproducts of mitochondrial respiration, into molecular oxygen (O_2_) and hydrogen peroxide (H_2_O_2_) (Abreu & Cabelli, 2010 ▸). As the only known mitochondrial enzyme capable of converting reactive 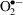 radicals into diffusible products that can be further metabolized or exported, MnSOD serves as the critical first line of defence against oxidative stress (Bresciani *et al.*, 2015 ▸; Grujicic & Allen, 2025 ▸). Unsurprisingly, dysregulation of MnSOD activity is associated with various human diseases, highlighting the continued need for a detailed structural understanding of its catalytic mechanism.

Human MnSOD physiologically functions as a homotetramer, with each active site containing a manganese ion, which cycles between oxidized (trivalent, Mn^3+^, pink) and reduced (divalent, Mn^2+^, colourless) states to drive two half-reactions to complete the catalytic cycle (Borgstahl *et al.*, 1992 ▸; Fee & Bull, 1986 ▸; Hsu *et al.*, 1996 ▸):
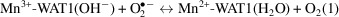

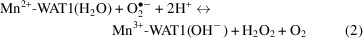


In the first half-reaction (equation 1), resting-state Mn^3+^ oxidizes 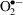 to the first reaction product, O_2_, which diffuses out of the mitochondria or becomes reabsorbed into the mitochondrial electron-transport chain. During this half-reaction, the active-site manganese is simultaneously reduced, and its WAT1 ligand receives a proton and becomes a water molecule (Azadmanesh *et al.*, 2021 ▸). In the second half-reaction (equation 2) Mn^2+^ binds a second 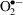 radical, and protons and electrons are again transferred simultaneously to form H_2_O_2_ and to redox-cycle the active-site manganese. In this way, both half-reactions use proton-coupled electron-transfer (PCET) events (Reece & Nocera, 2009 ▸) to rapidly convert 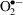 to O_2_ and H_2_O_2_.

Decades of extensive structural characterization efforts have yielded a total of 39 human MnSOD structures in the Protein Data Bank (PDB). Out of them, almost all reported H_2_O_2_-soaked MnSOD structures have consistently identified H_2_O_2_ bound to the catalytic manganese, in a near side-on configuration, that replaces the WAT1 solvent (Azadmanesh *et al.*, 2024 ▸, 2025 ▸; Porta *et al.*, 2010 ▸). This metal-ligated H_2_O_2_-binding site is typically referred to as the LIG position. A recent H_2_O_2_-soaked Trp161Phe MnSOD neutron structure identified a second H_2_O_2_-binding site, named PEO, between the second-sphere gateway residues Tyr34 and His30 at the opening of the active-site solvent funnel (Azadmanesh *et al.*, 2024 ▸). Tyr34 and His30 are important for the PCET reaction of native MnSOD, as they directly participate in critical proton transfers during catalysis (Azadmanesh *et al.*, 2021 ▸, 2025 ▸). This is shown by the significantly reduced kinetics whenever the Tyr34- and His30-mediated proton transfers are disrupted (Guan *et al.*, 1998 ▸; Hearn *et al.*, 2003 ▸; Hsu *et al.*, 1996 ▸; Quint *et al.*, 2006 ▸). While these structures and the kinetic studies offer valuable insights into catalytically important active-site residues, as well as the H_2_O_2_-binding modes in MnSOD, to date no electron density for H_2_O_2_ has been observed beyond the PEO binding site on the solvent-exposed side of the active-site funnel. Moreover, under specific conditions MnSOD switches to a slower catalytic model, known as the MnSOD product-inhibited pathway, which proceeds via the rate-limiting resolution of a dead-end catalytic intermediate, followed by slow H_2_O_2_ release. Even in severely product-inhibited MnSOD variants, such as Trp161Phe (Azadmanesh *et al.*, 2024 ▸; Cabelli *et al.*, 1999 ▸) and Tyr34Phe (Azadmanesh *et al.*, 2025 ▸; Guan *et al.*, 1998 ▸; Hearn *et al.*, 2001 ▸; Ramilo *et al.*, 1999 ▸), which release peroxide slowly, we do not observe any evidence of H_2_O_2_ binding beyond the MnSOD solvent gate. These findings suggest that once H_2_O_2_ crosses the solvent gateway, it rapidly diffuses into bulk solvent.

Further complicating structural efforts, partially occupied and/or mobile H_2_O_2_ molecules are often indistinguishable from water (H_2_O) in medium- to low-resolution X-ray electron-density maps, particularly in the absence of neutron diffraction data that can accurately resolve hydrogen positions. Thus, the following key questions remain unresolved. (i) What is the fate of H_2_O_2_ following release from the PEO site? (ii) Are there any transient H_2_O_2_-binding interactions occurring beyond the Tyr34–His30 solvent gateway? (iii) What governs H_2_O_2_ diffusion on the solvent-exposed surface of MnSOD? These questions are important because MnSOD catalysis is limited not by the chemical transformation itself, but by the diffusion of substrate and product through the ∼10 Å deep, ∼5 Å wide active solvent cavity (Azadmanesh *et al.*, 2017 ▸)

**Figure 1:**
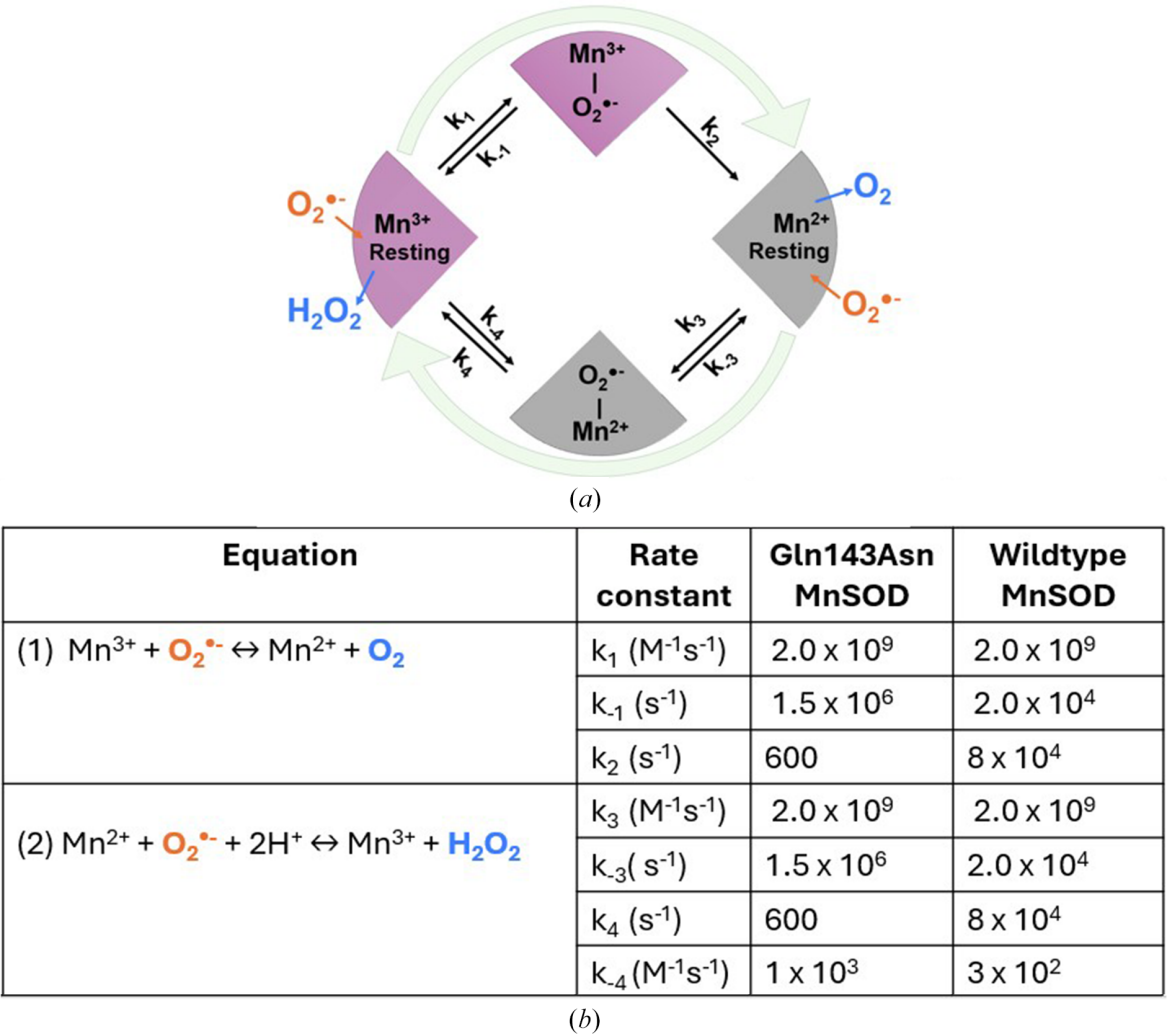
The kinetics of MnSOD catalysis. (*a*) MnSOD reaction scheme. (*b*) Kinetic parameters of human wild-type and Gln143Asn MnSOD enzymes. Data from Hsieh *et al.* (1998 ▸) and Guan *et al.* (1998 ▸).

The goal of this current study is to identify all possible H_2_O_2_-binding sites on the solvent-exposed side of the MnSOD solvent funnel by using a kinetically slow and catalytically impaired Gln143Asn MnSOD variant in which a second-sphere residue that plays a critical role in the PCET reaction mechanism of native MnSODs is mutated (Hsieh *et al.*, 1998 ▸; Lévêque *et al.*, 2000 ▸). Azadmanesh and coworkers showed that in the first half-reaction, upon chemical reduction of Mn^3+^ to Mn^2+^, Gln143 donates a proton to the active-site metal-bound solvent (WAT1-OH^−^) to generate WAT1-H_2_O (equation 1). The WAT1-OH^−^ negative charge is thought to have stabilized the positive charge of Mn^3+^ in the oxidized state. Presumably, in the second half of the reaction (equation 2), Gln143 takes the proton back from WAT1-H_2_O, regenerating the WAT1-OH^−^ negative charge simultaneously as Mn^2+^ is oxidized to Mn^3+^. Through this critical and reversible proton-transfer event, Gln143 is directly coupled to the redox cycling of the catalytic metal in MnSOD that drives its catalysis (Azadmanesh *et al.*, 2021 ▸). Substituting Gln143 with Asn preserves the active-site architecture (Hsieh *et al.*, 1998 ▸), but the hole created disrupts proton transfers with the WAT1 solvent. Consequently, redox cycling of the catalytic manganese is slowed, as shown by an over ∼130-fold reduction in *k*_cat_ (wild-type *k*_cat_ = 40 ms^−1^, Gln143Asn *k*_cat_ = 0.3 ms^−1^; Hsieh *et al.*, 1998 ▸; Lévêque *et al.*, 2000 ▸; Guan *et al.*, 1998 ▸; Fig. 1 ▸). Thus, the kinetic rate constants of Gln143Asn MnSOD have the overall effect of tending to keep the active-site manganese reduced. While this loss of catalytic efficiency compromises the overall function of Gln143Asn MnSOD, it offers a distinct experimental advantage for our study; the slowed turnover rate allows a greater control of experimental design, cryo-trapping and structural characterization of bound H_2_O_2_ molecules. Our results reveal many H_2_O_2_ molecules beyond the canonical LIG and PEO positions, spanning the space extending from the edge of the Tyr34–His30 solvent gateway to the bulk solvent. These findings expand the structural landscape of how H_2_O_2_ exits the MnSOD active site.

## 2. Materials and methods

### 2.1. Expression, purification and crystallization of Gln143Asn MnSOD

The Gln143Asn plasmid was ordered from GenScript and codon-optimized for expression in *Escherichia coli*. Following transformation of *E. coli* BL21(DE3) cells with the plasmid, a small culture was incubated at 37°C and 225 rev min^−1^ until visible turbidity was reached in Terrific Broth (TB) supplemented with 30 µg l^−1^ kanamycin. Next, 2 l of TB supplemented with 30 µg l^−1^ kanamycin and 16 ml 100% glycerol was inoculated with ∼5 ml of the small growth and incubated at 37°C with vigorous shaking at 225 rev min^−1^ until an OD_600_ of 0.8–1.0 was reached. A bolus of 8 m*M* MnCl_2_ and 1 m*M* isopropyl β-d-1-thiogalactopyranoside (IPTG) was then added and the flasks were incubated at 37°C and 140 rev min^−1^ overnight. Induced cells were harvested by centrifugation using a Sorval Lynx 6000 with a Fiberlite F9-6 × 1000 LEX rotor operated at 4°C and 8000 rev min^−1^ for 40 min. The pellets were stored at −20°C. For Gln143Asn MnSOD protein purification, pellets were thawed at room temperature and thoroughly resuspended in lysis buffer (10 m*M* MnCl_2_, 10 m*M* MOPS pH 7.8), followed by filtration and removal of the insoluble fraction. Next, the soluble fraction was lysed with an EmulsiFlex-C3 at a pressure equal to or greater than 138 MPa for ∼5 sample passes. The lysate was centrifuged at 4°C and 8000 rev min^−1^ for 40 min, and the supernatant was transferred to a hot-water bath at 55°C for 1 h. The solute was then centrifuged again, and the supernatant (containing the soluble proteins) was diluted using 25 m*M* MES pH 5.5 at a 1:2 protein:buffer ratio. The diluted protein was then loaded onto a HiPrep 16 × 10 CMFF cation-exchange column using an ÄKTApure HPLC (GE Healthcare). The sample was buffer-exchanged into 10 m*M* MES pH 5.5 on the column, followed by protein elution from the resin with 10 m*M* MES pH 6.5. Purification was confirmed by the presence of a single band corresponding to the correct molecular weight of 23 kDa on a 4–12% SDS–PAGE gel stained with Coomassie Blue. Before crystallization, all pure fractions were pooled together, buffer-exchanged into 0.25 m*M* potassium phosphate pH 7.8 using a 10 kDa concentrator (Millipore, Sigma–Aldrich) and concentrated to ∼23 mg ml^−1^ (∼1 m*M*).

24-well hanging-drop trays were set up with a 1:1 ratio of concentrated protein solution and reservoir solution consisting of 1.8 *M* potassium phosphate pH 7.8 and incubated at room temperature. Gln143Asn MnSOD crystallized in the high-symmetry space group *P*6_1_22, and the crystals grew to final sizes of ∼100 µm in length in a week. The crystal trays were hand-carried by flight to the Stanford Synchrotron Radiation Lightsource (SSRL) for data collection.

### 2.2. X-ray crystallographic data collection, processing, refinement and validation

A single crystal of Gln143Asn MnSOD was exchanged into cryo-solution (2.5 *M* potassium phosphate pH 7.8) at room temperature, manually plunged into liquid nitrogen and then mounted with a 100 µm-sized cryo-loop (MiTeGen) on SSRL beamline 14-1. The mounted crystal was annealed by manually blocking the cryo-stream for 5 s. For H_2_O_2_ soaking, a 30% stock solution of H_2_O_2_ (Millipore Sigma) was diluted 100-fold and pipetted into the drop of mother liquor containing the single Gln143Asn crystal to reach a final H_2_O_2_ concentration of 0.3% *in crystallo*. The total soaking time was ∼30 s at room temperature, and was followed by quick plunging into liquid nitrogen to cryo-trap the soaked H_2_O_2_ in the crystal, mounting and annealing as for the resting-state Gln143Asn MnSOD crystal. The process is schematized in Supplementary Fig. S1. X-ray crystallographic data for both resting-state and H_2_O_2_-treated Gln143Asn MnSOD were collected at 100 K using a Dectris PILATUS 16M pixel-array detector and a controllable axial nitrogen cryo-stream (Oxford Cryosystems). Data-collection parameters are summarized in Table 1 ▸. Initial phases were obtained using the *Phaser* molecular-replacement (*Phaser-MR*) package within the *Phenix* software suite (Liebschner *et al.*, 2019 ▸), with the wild-type MnSOD structure (PDB entry 7klb; Azadmanesh *et al.*, 2021 ▸) serving as the phasing model. Model building was performed with *Coot* (Emsley *et al.*, 2010 ▸) followed by iterative rounds of refinement cycles using *phenix.refine*. All refinement strategies were based on maximum-likelihood-based target functions and included the refinement of (i) individual atomic coordinates (in both reciprocal and real space) and (ii) individual isotropic atomic displacement parameters (ADPs) for all atoms, with automatic weight optimization. Only riding hydrogens were added to the models (Liebschner *et al.*, 2020 ▸) and appropriate metal-coordination restraints were generated using idealized geometries with the *ReadySet* tool. Gln143Asn, like the wild-type enzyme, crystallizes as a homodimer, with monomers *A *and *B* in the asymmetric unit, and crystallographic symmetry operations were applied to generate the physiological tetramer using the *PyMOL* Molecular Graphics System (Schrödinger). Final model validations were performed using *MolProbity* (Chen *et al.*, 2010 ▸). Coordinate error estimates, as calculated with *phenix.refine*, are reported in Table 1 ▸ as a measure of model precision. Coordinates and structure factors for the resting-state and H_2_O_2_-treated Gln143Asn MnSOD X-ray crystal structures have been deposited in the Protein Data Bank (PDB) under accession codes 9nr0 and 9nsj, respectively.

### 2.3. Metal center-specific electrostatic surface generation

To generate solvent-excluded electrostatic surfaces of tetrameric MnSOD proteins that accurately reflect the electronic charge distribution, spin multiplicity and redox potential of the active-site manganese centres, we implemented a three-step protocol to account for electronic effects imparted by the primary coordination sphere (PCS) ligands (Van Stappen *et al.*, 2022 ▸), as schematized in Supplementary Fig. S2.

*Step 1.* Initial per-residue charges were assigned using the *PDB*2*PQR* (Dolinsky *et al.*, 2004 ▸, 2007 ▸) web service hosted at https://www.poissonboltzmann.org/. This generated PQR files containing (i) atomic partial charges of all residues, computed using the AMBER force field (Hornak *et al.*, 2006 ▸), and (ii) protonation states of ionizable residues, based on p*K*_a_ values predicted by *PROPKA* 3.0 at pH 7.0 (Olsson *et al.*, 2011 ▸).

*Step 2.* To determine the electronic properties of the catalytic manganese in specific redox and spin states, the protein structures were truncated to only retain the first-shell ligands coordinating the manganese ion, thereby preserving the key geometric and electronic features of the metal site. Density-functional theory (DFT) calculations were performed on the isolated metal centres, using the high-spin sextet state for the divalent manganese ion in the Gln143Asn enzyme, according to established protocols in Neves *et al.* (2013 ▸) and Azadmanesh *et al.* (2017 ▸). Single-atomic electrostatic potential (ESP) charges for the manganese ion and its direct ligands were derived using the Merz–Kollman scheme and subsequently refined using the restrained electrostatic potential (RESP) methodology. As a benchmark, we applied the same DFT protocol to the five-coordinate, high-spin quintet resting state of the wild-type Mn^3+^SOD enzyme (PDB entry 5vf9; Azadmanesh *et al.*, 2017 ▸).

*Step 3.* The ESP-refined metal charges were then integrated with the remainder of the PQR-derived charges of the tetrameric protein to compute full electrostatic surface maps using the *Adaptive Poisson–Boltzmann Solver* (*APBS*) electrostatics plugin within *PyMOL*, following the established protocols (Azadmanesh *et al.*, 2017 ▸).

### 2.4. Mn *K*-edge HERFD-XANES spectroscopy data collection, processing and analysis

A solution of 3 m*M* Gln143Asn MnSOD (∼70 mg ml^−1^) in 25 m*M* potassium phosphate pH 7.8 was treated with 280 m*M* [1%(*w*/*v*)] H_2_O_2_ to isolate the H_2_O_2_-bound state of the Gln143Asn variant. Mn *K*-edge HERFD-XANES spectra (Proux *et al.*, 2017 ▸) were recorded using a liquid-helium cryostat at 10 K on beamline 15-2 of SSRL. The incident energy was tuned to the first derivative of an internal manganese foil at 6539 eV. X-ray irradiation was carefully monitored so that two subsequent scans of the same spot did not have photoreduction differences, and different spots along samples were scanned. When appropriate, aluminium foil was inserted into the beam path to attenuate the incident flux. For HERFD-XANES measurements, a Johann-type hard X-ray spectrometer with six manganese analyser crystals was used with a liquid-nitrogen-cooled Si(311) double-crystal monochromator. Experimental spectra were processed, visualized and plotted using the *Larch* software package. The post-edge regions were normalized, and pre-edge peak fittings were performed using pseudo-Voigt functions. Pre-edge intensities were quantified as the integrated area under the fitted curves.

## 3. Results and discussion

### 3.1. Multiple H_2_O_2_-binding sites were revealed beyond the Tyr34–His30 solvent gateway in Gln143Asn MnSOD

**Figure 2:**
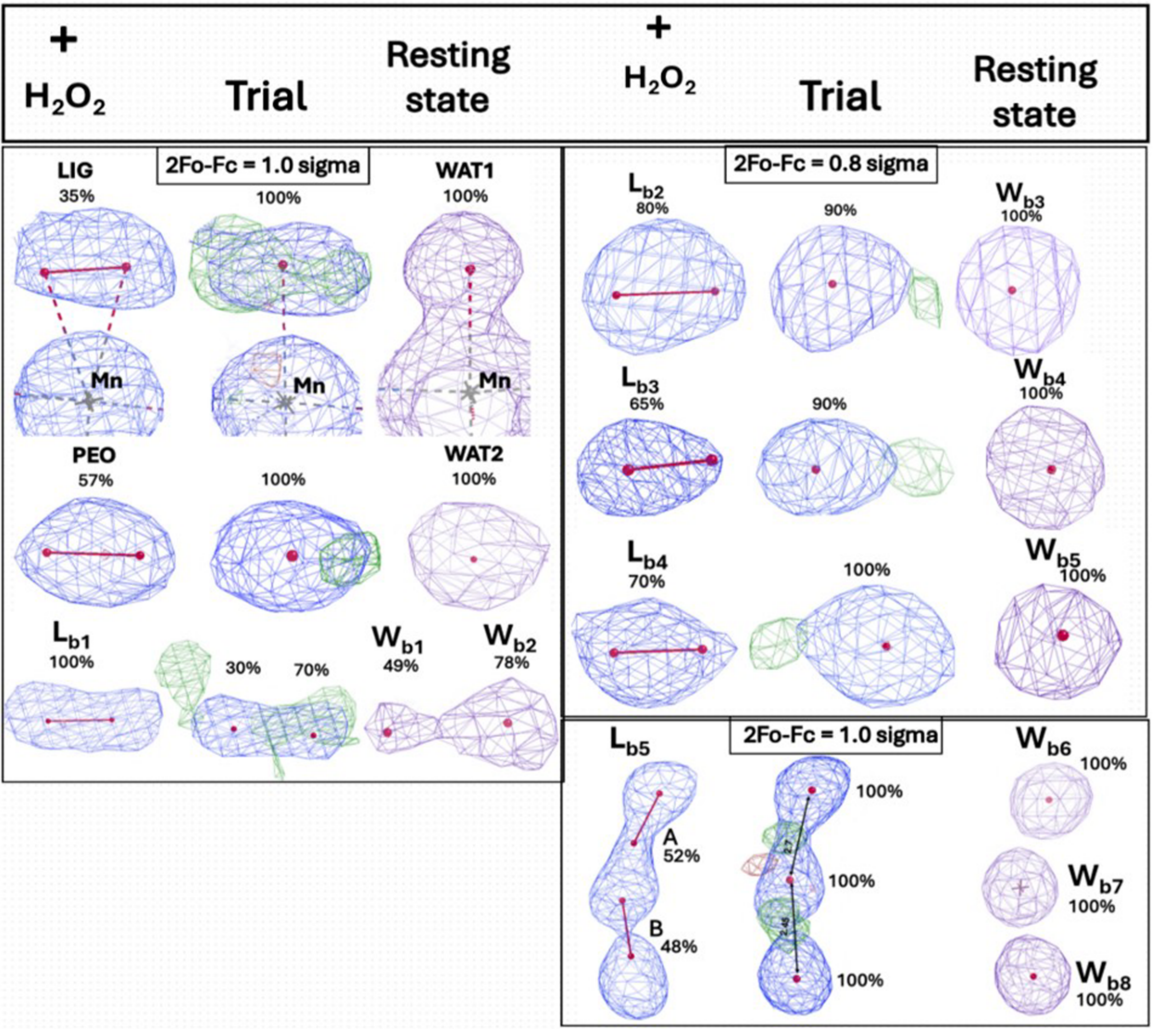
Electron-density maps of the modelled peroxides. The left panel shows 2*F*_o_ − *F*_c_ electron densities for all of the peroxide molecules modelled in the treated Gln143Asn structure (blue mesh) at the indicated contour levels. To validate this, we ran refinement trials where each L_b_ was replaced by a fully occupied water (middle panel) as in the resting-state structure (right panel). The *F*_o_ − *F*_c_ difference densities (green mesh, contoured to 3.0σ) in the middle trial panels indicate the need to model two O atoms in the treated structure, rather than one as in the resting state. Moreover, the 2*F*_o_ − *F*_c_ density is elongated for the L_b_ sites in the treated structure (blue mesh), whereas it appears more spherical in the resting state (purple mesh).

In this present study, we report two high-resolution X-ray crystal structures of the Gln143Asn MnSOD enzyme: (i) a 1.33 Å resolution structure of the H_2_O_2_-treated enzyme (PDB entry 9nr0) and (ii) a 1.55 Å resolutiom structure of the untreated resting-state enzyme (PDB entry 9nsj) (Table 1 ▸). In addition to H_2_O_2_ binding at the canonical LIG and PEO sites in the treated Gln143Asn enzyme, our results reveal a striking accumulation of five distinct cryo-trapped H_2_O_2_ molecules, either fully or partially occupying the space between the solvent-exposed enzyme surface and the Tyr34–His30 solvent gate. These new bulk-exposed peroxides, named L_b_ for Lig_bulk_ binding sites, identified in this study have never been observed in any other peroxide-soaked MnSOD. Superposition of the electron densities of the modelled L_b_ peroxides in the treated structure (Fig. 2 ▸, left, blue mesh) with those of the corresponding waters in the resting-state structure (Fig. 2 ▸, right, purple mesh) shows that one or both O atoms of the L_b_ H_2_O_2_ overlap with either fully or partially occupied water molecules in the untreated resting-state structure. To validate that the observed L_b_ densities truly correspond to H_2_O_2_ molecules, rather than misassigned or mobile waters, we performed a trial refinement in which we replaced each L_b_ peroxide in the treated structure with a fully occupied water molecule, as in the resting state. However, this does not satisfy the electron density, as shown by the appearance of positive *F*_o_ − *F*_c_ difference density around one of the missing O atoms in the trial structure (Fig. 2 ▸, middle, green mesh). Such a large number of H_2_O_2_-binding sites have never been observed in any previous MnSOD structure, and provide strong validation for our chosen experimental strategy, which leverages the slowed catalysis of the Gln143Asn variant to reveal all potential H_2_O_2_-binding sites in MnSOD.

**Figure 3:**
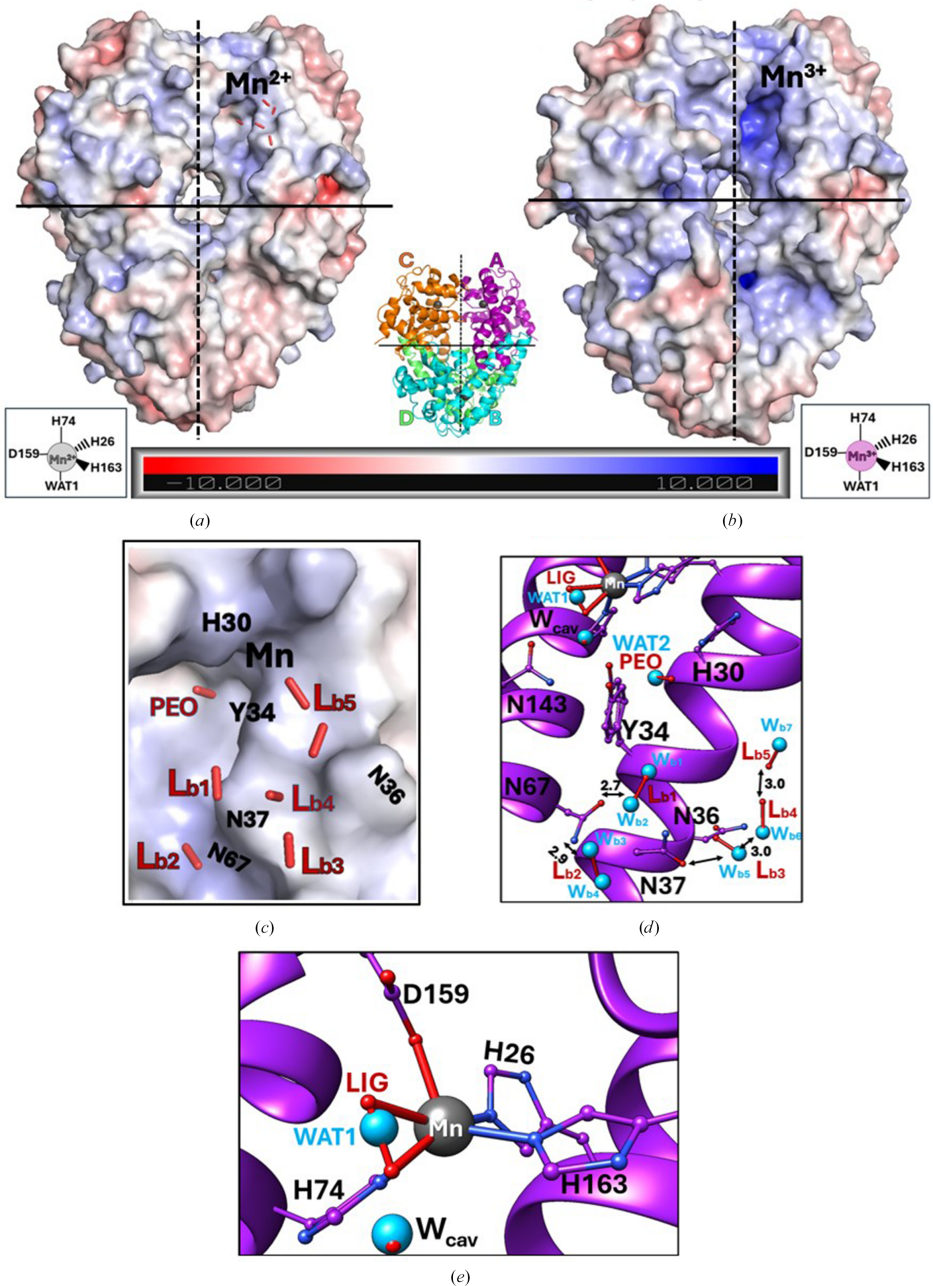
H_2_O_2_ binding in Gln143Asn MnSOD. (*a*, *b*) Resting-state Gln143Asn and wild-type MnSOD enzymes, overlaid on their respective electrostatic surfaces, with the location of the chain *A* active-site manganese labelled. All bulk-exposed L_b_ peroxides trapped in the Gln143Asn enzyme are shown as red sticks. Dimeric and tetrameric interfaces are marked by dashed and solid black lines, respectively. Insets show the pentavalent Mn^2+^ (grey sphere) and Mn^3+^ (pink sphere) ions and their primary coordination-sphere ligands in the Gln143Asn and wild-type enzymes, respectively. The middle inset shows the ribbon diagram of the tetrameric Gln143Asn MnSOD, with manganese ions shown as grey spheres. (*c*) Enlarged view of L_b_ H_2_O_2_ (red stick) on top of the displayed electrostatic surface. (*d*) All modelled H_2_O_2_, the active site (top) and the Tyr34–His30 solvent gate with the resting-state water molecules (blue spheres). (*e*) Enlarged view of the active site of Gln143Asn, with bound LIG (red sticks) peroxide in the treated structure, and the WAT1 solvent (blue spheres) and W_cav_.

Importantly, the L_b_ H_2_O_2_ molecules bind to the solvent-exposed surface of the Gln143Asn MnSOD tetramer (Figs. 3 ▸*a*–3 ▸*c*). We note that the resting-state electrostatic solvent-accessible surface of Gln143Asn MnSOD is different from the surface of resting-state wild-type MnSOD. The active-site funnel of the wild type is much more positively charged than that of the Gln143Asn mutant. This is because the wild-type resting state contains Mn^3+^ and the Gln143Asn resting state contains Mn^2+^. This indicates that the redox cycling by wild-type MnSOD during catalysis is supported by the cycling of its electrostatic surface between positive and more neutral surfaces. Gln143Asn MnSOD retains the more neutral surface most of the time, and this seems to help stabilize the H_2_O_2_-binding sites. Figs. 3 ▸(*d*) and 3 ▸(*e*) show enlarged views of all modelled peroxides in the treated Gln143Asn structure (red sticks) overlaid on top of the corresponding waters in the resting state (blue spheres).

**Figure 4:**
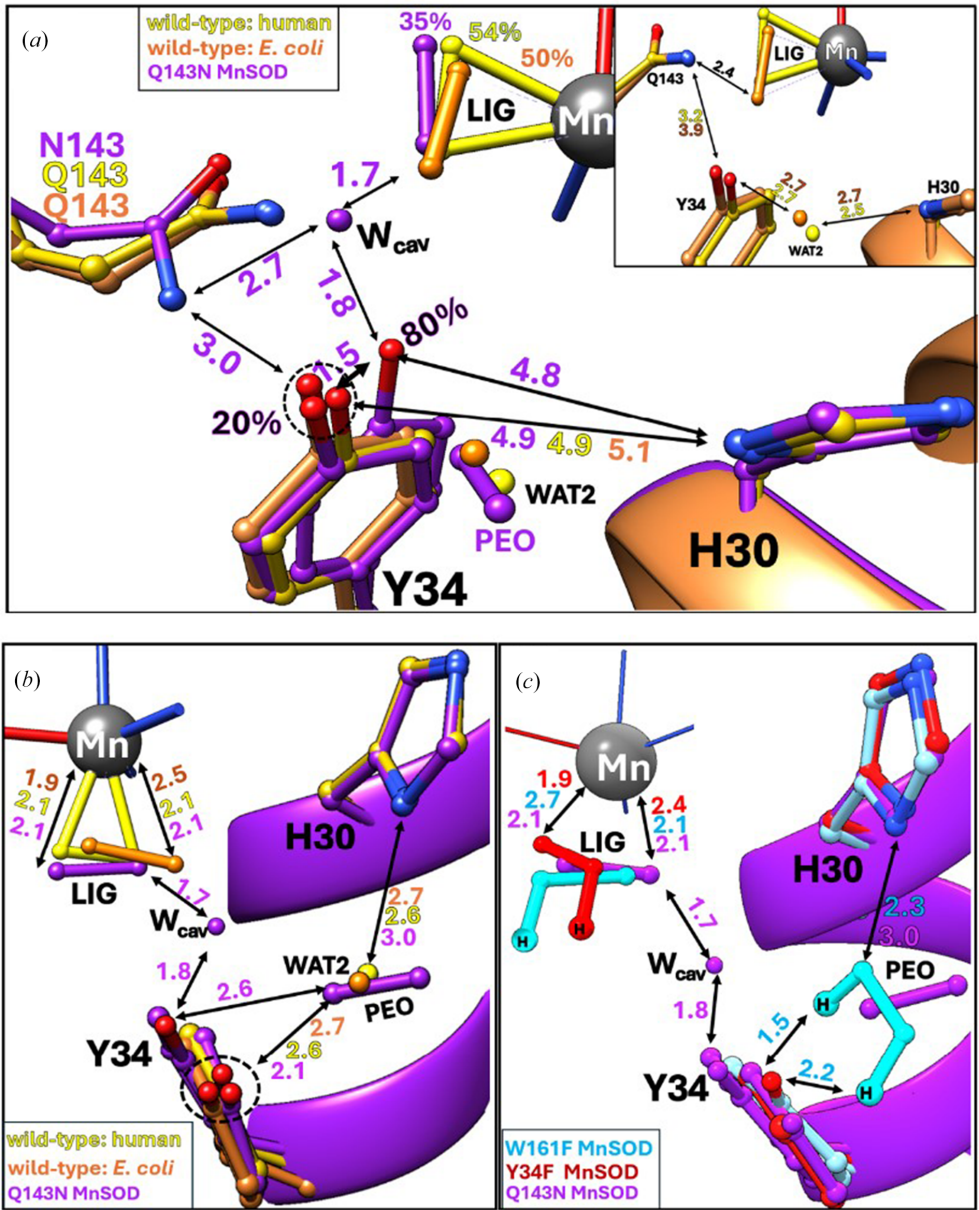
Comparison with known H_2_O_2_-soaked MnSOD structures in the PDB. Structural comparisons of H_2_O_2_-treated human Gln143Asn MnSOD (purple) with (*a*) human (yellow; PDB entry 8vj5) and (*b*) *E. coli* (orange; PDB entry 3k9s) wild-type enzymes, as well as with (*c*) the product-inhibited MnSOD variants Trp161Phe (cyan, neutron, PDB entry 8vhw) and Tyr34Phe (red, neutron, PDB entry 9bvy). We observe H_2_O_2_ binding in the pre-established LIG and PEO binding sites, with subtle differences, potentially owing to the Gln143 mutation-induced cavity. Notably, Tyr34 samples a shifted conformation (∼80% occupancy) in this cavity, drawing closer to both the LIG H_2_O_2_ and His30. This conformational flexibility is not typically observed in wild-type or other variants lacking this mutation-induced cavity between the metal primary sphere and the second-shell solvent gateway.

In addition to the L_b_ H_2_O_2_ molecules on the solvent-exposed surface, we observe H_2_O_2_ binding at the metal-ligated LIG position and at the PEO position at the mouth of the active-site solvent-funnel gateway between second-shell residues Tyr34 and His30. Overall, H_2_O_2_ treatment does not appear to significantly alter the active-site architecture of Gln143Asn MnSOD, except for the partial displacement of the manganese-bound solvent (WAT1) by a partially occupied (∼35%) H_2_O_2_ molecule bound at the LIG position, opposite His26 (Figs. 3 ▸*e* and 4 ▸). Superposition with all other previously published structures of H_2_O_2_-treated human MnSODs, *i.e.* wild type (PDB entry 8vj5; Figs. 4 ▸*a* and 4 ▸*b*, yellow), Trp161Phe (Fig. 4 ▸*c*, cyan) and Tyr34Phe (Fig. 3 ▸*c*, red), as well as *E. coli* wild-type MnSOD (PDB entry 3k9s; Figs. 4 ▸*a* and 4 ▸*b*, orange), shows that H_2_O_2_ binds the active-site LIG position in the Gln143Asn variant (Figs. 4 ▸*a*–4 ▸*c*, purple) in a configuration very similar to the previously observed nearly side-on binding modes in the human MnSOD variants, rather than end-on, as seen in the *E. coli* wild-type MnSOD.

Additionally, we observe a 57% occupied H_2_O_2_ near the PEO binding site, which has so far only been observed in the H_2_O_2_-soaked neutron structure of Trp161Phe MnSOD (PDB entry 8vhw; Azadmanesh *et al.*, 2024 ▸) at full occupancy (Fig. 4 ▸*c*, cyan). Superposition of the two H_2_O_2_-bound MnSOD structures reveals that H_2_O_2_ binds at the PEO site of the Gln143Asn variant (Fig. 4 ▸*c*, purple) in a slightly different configuration. However, we do not know what causes this change in H_2_O_2_ binding at the PEO site of Gln143Asn MnSOD.

### 3.2. Peroxide binding subtly alters the active-site electrostatics of Gln143Asn MnSOD without changing the redox or coordination states of the catalytic manganese

**Figure 5:**
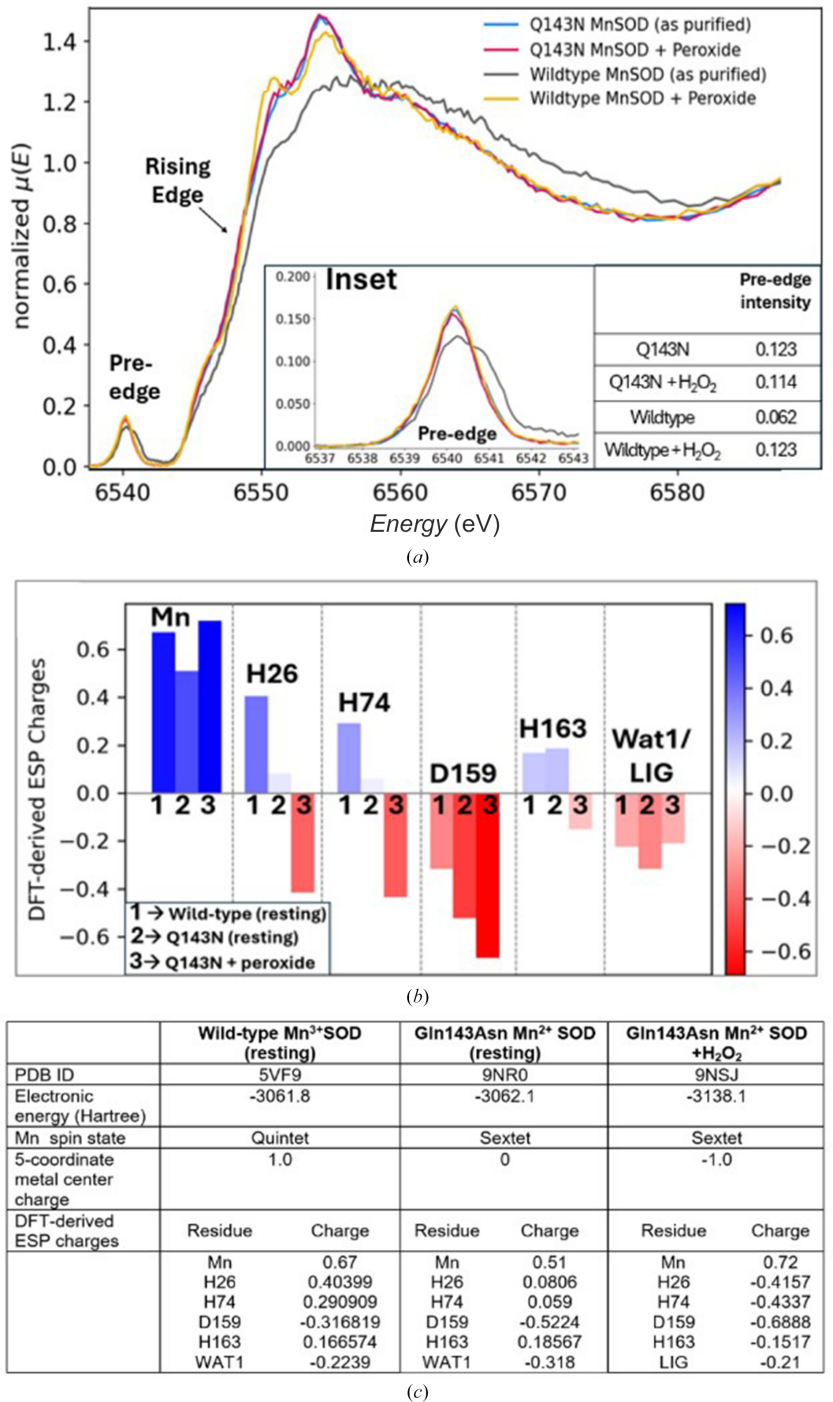
HERFD-XANES spectroscopy. (*a*) Normalized Mn *K*-edge HERFD-XANES spectra of wild-type and Gln143Asn MnSOD enzymes both in resting and H_2_O_2_-soaked forms. The inset shows a magnified view of the pre-edge region (6537–6543 eV), along with their individual pre-edge intensities. (*b*, *c*) Manganese redox state and/or H_2_O_2_ treatment affects the individual DFT-derived ESP charges of the manganese primary coordination-sphere residues.

To monitor changes in the oxidation state and coordination geometry of the catalytic manganese in the Gln143Asn variant upon H_2_O_2_ treatment, we compared the Mn *K*-edge high-energy-resonance fluorescence-detected X-ray absorption near-edge spectra (HERFD-XANES) of the purified enzyme before and after H_2_O_2_ treatment, following established protocols (Azadmanesh *et al.*, 2024 ▸, 2025 ▸). We observe no detectable shift in either rising-edge or pre-edge features (Fig. 5 ▸*a*, blue versus red), indicating no alteration in the redox state or coordination geometry of the catalytic manganese after H_2_O_2_ treatment in Gln143Asn MnSOD. Notably, the spectral features of both Gln143Asn samples closely resemble those of the H_2_O_2_-treated wild-type MnSOD (Fig. 5 ▸*a*, yellow), which has been previously established to contain the reduced Mn^2+^ ion in its active site (Azadmanesh *et al.*, 2024 ▸). The pre-edge intensities of both Gln143Asn samples are comparable to each other and to the H_2_O_2_-treated wild-type enzyme, indicating highly similar metal centres across all three conditions. In contrast, the spectrum of the resting-state wild-type MnSOD (Fig. 5 ▸*a*, black) shows a clear shift of both rising-edge and pre-edge features towards higher energies, consistent with the established presence of an oxidized Mn^3+^ metal in the resting wild-type MnSOD.

DFT calculations predict no significant change in the charge or spin state of the catalytic Mn^2+^ in the Gln143Asn enzyme upon H_2_O_2_ treatment. However, certain trends emerge (Figs. 5 ▸*b* and 5 ▸*c*): (i) the charges of both His26 and His74 switch sign, going from positive to negative (and closer to the wild-type resting-state values), (ii) Asp159 is less negatively charged in the resting state of the Gln143Asn enzyme compared with the wild type, but becomes more negative with H_2_O_2_ addition, and (iii) His163 flips from negatively to positively charged when comparing the wild type with the variant, indicating potential protonation-state change. However, these conclusions remain to be experimentally validated. Moreover, it is important to note that our DFT models treat LIG as a dioxygen species rather than H_2_O_2_ to account for the caveat that X-ray electron-density maps cannot pinpoint proton positions accurately. This is particularly relevant considering recent neutron diffraction studies of H_2_O_2_-soaked Trp161Phe (Fig. 4 ▸*c*, cyan) and Tyr34Phe (Fig. 4 ▸*c*, red) Mn^2+^SODs, both of which stabilize a singly protonated dioxygen species, hydroperoxyl anion (**−**OOH), following H_2_O_2_-induced metal reduction (Azadmanesh *et al.*, 2024 ▸, 2025 ▸).

Overall, we conclude that H_2_O_2_ treatment does not alter the redox state or coordination geometry of the catalytic manganese in the Gln143Asn enzyme, unlike all other H_2_O_2_-treated MnSOD variants characterized previously. This further demonstrates how Gln143Asn is perpetually frozen in the reduced state without the necessary proton-transfer events. This loss of redox flexibility at the expense of catalytic turnover distinguishes Gln143Asn from all other spectrally characterized human MnSOD variants to date.

## 4. Conclusions

The high-resolution crystal structure of H_2_O_2_-treated human Gln143Asn MnSOD reported here reveals previously unobserved, L_b_ H_2_O_2_-binding sites on the solvent-exposed surface, in addition to the canonical LIG and PEO positions (Figs. 2 ▸, 3 ▸ and 4 ▸). This contrasts with all previously published H_2_O_2_-soaked MnSOD structures, which have captured H_2_O_2_ binding only within the first or second coordination shells. Although the fact that product release occurs too rapidly in the wild-type enzyme offers a plausible explanation as to why we do not catch transient H_2_O_2_ beyond the active-site gateway on the solvent-exposed side, their absence even in severely product-inhibited MnSOD variants, such as Trp161Phe and Tyr34Phe, suggests that the principal kinetic bottleneck for product release from MnSOD is H_2_O_2_ diffusion across the Tyr34–His30 solvent gate. It appears that once beyond this point, H_2_O_2_ escapes rapidly into bulk solvent, evading experimental detection methods. To overcome this gap, we leveraged the slow kinetics of the catalytically impaired Gln143Asn MnSOD to successfully stabilize several L_b_ H_2_O_2_ beyond the active-site solvent funnel, thus depicting a more extensive MnSOD H_2_O_2_-interaction landscape than previously available (Fig. 3 ▸). The redox cycling of the active-site metal in Gln143Asn MnSOD is impaired; this causes the electrostatic surface to stay in a more neutral condition and the H_2_O_2_ molecules are immobilized (Figs. 3 ▸*a* and 3 ▸*b*). These solvent-exposed L_b_ H_2_O_2_ molecules likely represent a native ensemble of product molecules diffusing across the active-site solvent channel, extending from the gateway at the second shell to the bulk-solvent-exposed surface. This phenomenon has never been structurally visualized before in any H_2_O_2_-soaked MnSOD enzyme.

Tyr34 in Gln143Asn MnSOD adopts a dominantly shifted conformation (∼80% occupancy), even in the absence of H_2_O_2_ soaking (Figs. 3 ▸ and 4 ▸). This conformation is stabilized by an additional solvent molecule positioned in the mutation-induced cavity, namely W_cav_. This stabilizing water has also been reported in the previously published Gln143Asn resting-state structure (PDB entry 1qnm; Hsieh *et al.*, 1998 ▸). Interestingly, similar new cavity waters have been identified in the active sites of other MnSOD variants, even at significantly lower data resolutions (<2.0 Å). These include Gln143Ala (PDB entry 1em1; Lévêque *et al.*, 2000 ▸) and Trp161Ala (Hearn *et al.*, 2001 ▸). These new cavity waters subtly change the hydrogen-bonding environments within the active site of MnSOD, without any significant change in their overall structures. The W_cav_ solvent modelled in the Gln143Asn cavity of the peroxide-treated enzyme orients toward the LIG position-bound species, creating a tight interaction network that could stabilize bound H_2_O_2_ at the LIG site (Table 2 ▸). This network is not observed in the wild-type or other MnSOD variants, due to steric hindrance from Gln. This water molecule might be able to donate a proton to WAT1, and this may be why Gln143Asn MnSOD retains some activity. The alternate Tyr34 conformation and the presence of the W_cav_ solvent introduce new hydrogen bonds within the active site of Gln143Asn MnSOD, which are not observed in the wild-type enzyme (Supplementary Fig. S6). These may contribute to the slightly higher thermal stability of Gln143Asn MnSOD (∼1.8°C) reported previously (Hsieh *et al.*, 1998 ▸; Guan *et al.*, 1998 ▸).

HERFD-XANES and DFT analyses show no detectable change in the oxidation state, coordination number or spin state of the catalytic Mn^2+^ ion of Gln143Asn upon H_2_O_2_ treatment (Fig. 5 ▸*a*). These results confirm that this MnSOD variant remains stuck in a reduced, catalytically inert state, unable to drive the PCET-mediated MnSOD catalytic cycle. By decoupling proton transfer from catalysis, the Gln143Asn mutation stabilizes transient H_2_O_2_-bound states and opens a rare structural window into otherwise elusive steps of MnSOD catalysis. This work underscores the value of catalytically impaired mutants not as dysfunctional models but as powerful structural probes for revealing elusive mechanistic events in metalloenzymes.

## Supplementary Material

PDB reference: Gln143Asn MnSOD, untreated, 9nr0

PDB reference: treated with H_2_O_2_, 9nsj

Supplementary Table and Figures. DOI: 10.1107/S2053230X25009045/rl5204sup1.pdf

## Figures and Tables

**Table 1:** X-ray data-collection and refinement statistics Values in parentheses are for the outermost resolution shell.

	Gln143Asn MnSOD (untreated)	Gln143Asn MnSOD + H_2_O_2_
PDB code	9nr0	9nsj
Resolution range (Å)	37.14–1.55 (1.606–1.551)	39.34–1.33 (1.378–1.330)
Space group	*P*6_1_22	*P*6_1_22
*a*, *b*, *c* (Å)	78.20, 78.20, 237.21	78.11, 78.11, 236.02
α, β, γ (°)	90, 90, 120	90, 90, 120
Data-collection temperature (K)	100	100
Total No. of reflections	125342 (11951)	196692 (19149)
No. of unique reflections	62859 (6011)	98359 (9587)
No. of refinement reflections	62854 (6011)	98339 (9577)
Multiplicity	2.0 (2.0)	2.0 (2.0)
Completeness (%)	99.43 (97.25)	99.89 (99.18)
Mean *I*/σ(*I*)	6.85 (1.02)	9.38 (1.17)
Wilson *B* factor (Å^2^)	21.12	9.44
*R*_merge_	0.02582 (0.4213)	0.04186 (0.6241)
*R*_meas_	0.03652 (0.5958)	0.05920 (0.8826)
CC_1/2_	0.999 (0.634)	0.998 (0.541)
*R*_work_	0.1756 (0.3296)	0.1581 (0.3124)
*R*_free_	0.2148 (0.3660)	0.1942 (0.3034)
R.m.s.d., bond lengths (Å)	0.010	0.012
R.m.s.d., angles (°)	0.97	1.15
Coordinate error (Å)	0.19	0.15
Ramachandran favoured (%)	97.95	97.45
Average *B* factor (Å^2^)	26.24	16.33
Solvent content (%)	46.94	45.41

**Table 2:** Active-site water (W_cav_) coordination in resting-state and H_2_O_2_-treated Gln143Asn MnSOD Distances for chain *B* are in parentheses.

	Gln143Asn MnSOD, untreated (Å)	Gln143Asn MnSOD with H_2_O_2_ (Å)
PDB code	9nr0	9nsj
O^H^(Tyr34)–O(W_cav_)	2.01 (2.10)	1.83 (1.94)
N^δ2^(Asn143)–O(W_cav_)	2.79 (2.47)	2.66 (2.52)
O(WAT1)–O(W_cav_)	2.24 (2.35)	2.41
