# Supplementary material for: High-resolution X-ray structure of Gln143Asn manganese superoxide dismutase captures multiple hydrogen peroxide-binding sites
Source: Acta Crystallogr F Struct Biol Commun. 2025 Oct 23;81(Pt 11):467–77. doi: 10.1107/S2053230X25009045 (PMC12576686; doi:10.1107/S2053230X25009045)

STRUCTURAL BIOLOGY
COMMUNICATIONS

Volume 81 (2025)

Supporting information for article:

High-resolution X-ray structure of Gln143Asn manganese superoxide dismutase captures multiple hydrogen peroxide binding sites

Medhanjali Dasgupta, Katelyn Slobodnik, Erika A. Cone, Jahaun Azadmanesh, Thomas Kroll and Gloria E. O. Borgstahl

Table S1 Active site Mn bond lengths in resting-state and H_2O_2-bound Gln143Asn Mn^{2+} SOD and wildtype Mn^{3+} SOD.

	**Gln143Asn Mn^{2+}SOD (resting state) (Å)**	**Wt Mn^{2+}SOD (resting state) (PDB 7KLB) (Å)**
**PDB ID**	9NR0	7KLB
**Mn-$N^{\varepsilon 2}$(H26)**	2.14 (2.20)	2.18 (2.17)
**Mn-$N^{\varepsilon 2}$(H74)**	2.12 (2.15)	2.28 (2.16)
**Mn-$N^{\varepsilon 2}$(H163)**	2.24 (2.16)	2.25 (2.29)
**Mn-$O^{\delta 2}$(D159)**	2.04 (2.03)	2.09 (2.06)
**Mn-O(WAT1)**	2.12 (2.10)	2.31 (2.28)
	**Gln143Asn Mn^{2+} SOD with H_2O_2 (Å)**	**WtMnSOD (PDB 8VJ5) with H_2O_2 (Å)**
**PDB ID**	9NSJ	8VJ5
**Mn-$N^{\varepsilon 2}$(H26)**	2.21 (2.18)	2.16 (2.15)
**Mn-$N^{\varepsilon 2}$(H74)**	2.21 (2.17)	2.18 (2.18)
**Mn-$N^{\varepsilon 2}$(H163)**	2.18 (2.19)	2.21 (2.24)
**Mn-$O^{\delta 2}$(D159)**	2.03 (2.03)	2.07 (2.06)
**Mn-O(WAT1)**	2.16	___
**Mn-O_1(Lig)**	2.13 (2.09)	2.20 (2.10)
**Mn-O_2(Lig)**	2.19 (2.20)	2.19 (2.34)

Distances for chain B are in parentheses.

Figure S1 Cryoprotection protocol for Gln143Asn MnSOD crystals: The crystallization drop is shown as a red dashed circle with the final drop volume labeled in blue. The direction of red arrows shows if liquid is pipetted away from the crystal drop, or if cryo-buffer is pipetted into the drop. Only for the H_2O_2-treated Gln143Asn MnSOD dataset, 0.1 µL of a 30% stock of H_2O_2 (Sigma Aldrich) was added to a total drop volume of 10 µL to reach a final H_2O_2 concentration of 0.3% in the single crystal (step 11) and allowed to soak for ~30 seconds at room temperature. This was followed by plunging into liquid nitrogen and mounting in the cryo-stream at SSRL 14-1.

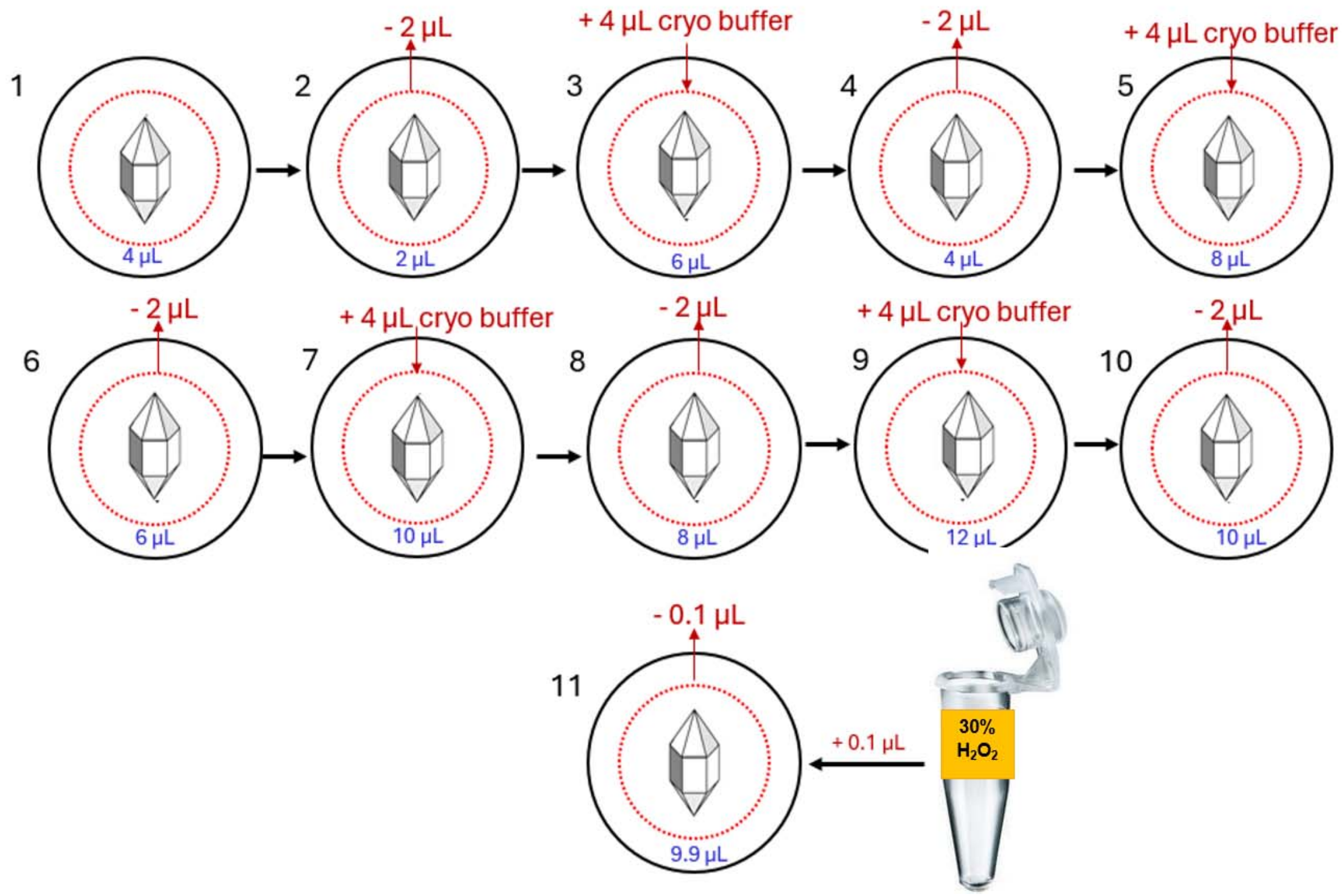

Figure S2 Workflow for generating metal-centered electrostatic surfaces.

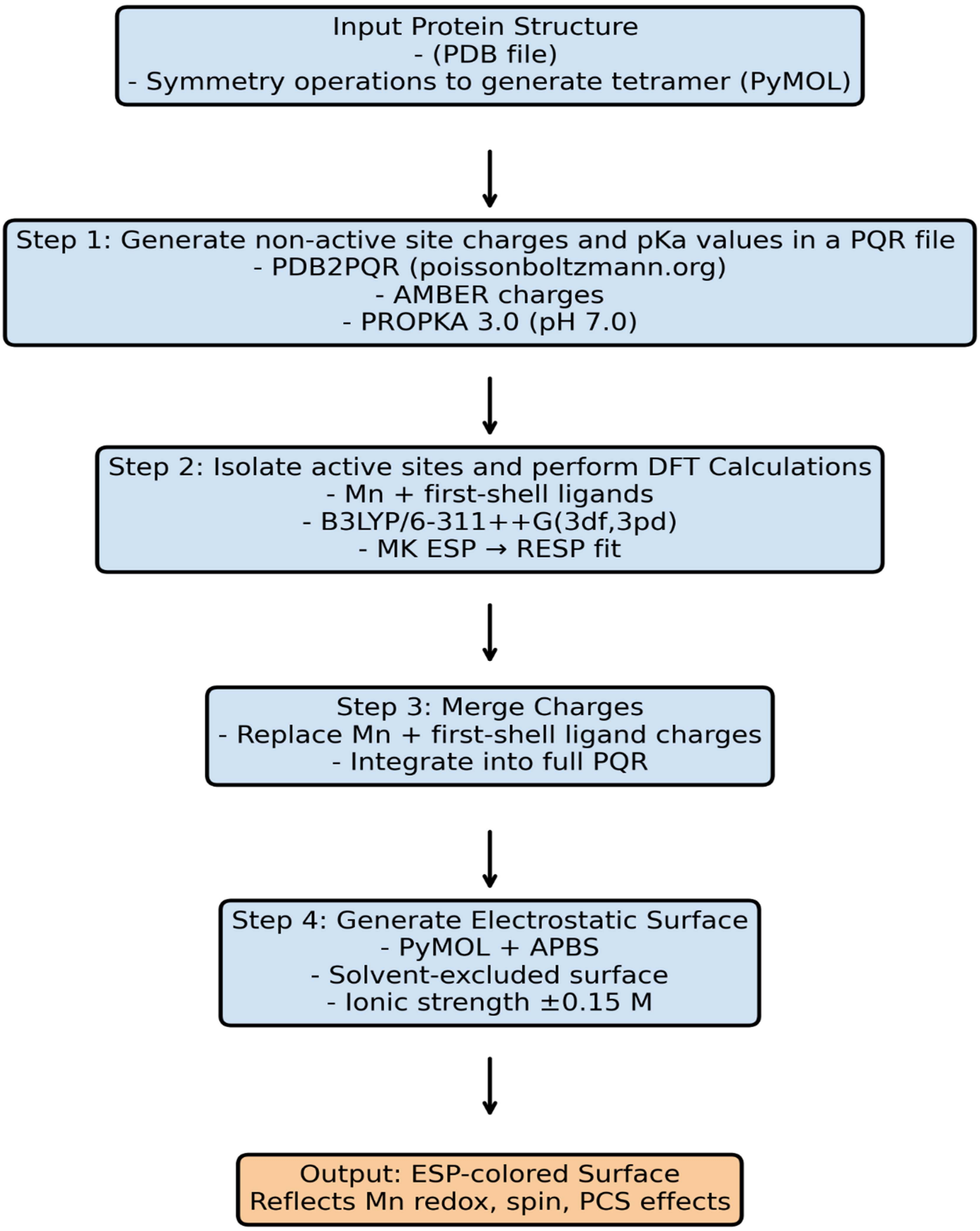

Figure S3 The Gln143 to Asn mutation creates a cavity adjacent to the active site which accommodates Tyr34 conformational flexibility in Gln143Asn MnSOD. (A) In the Gln143Asn MnSOD structure, the mutation-induced cavity accommodates a second conformation of Tyr34 (80% occupancy, circled in blue) and an additional water molecule, W_{CAV} (also circled in blue), resulting in new hydrogen bonding interactions. WAT2, located between Tyr34 and His30, is shown as a purple sphere. (B) The same view in reduced wild-type MnSOD (PDB ID: 7KLB) shows no evidence of a second Tyr34 conformation or W_{CAV} and has significantly fewer hydrogen bonds overall. These differences may contribute to the observed ~1.8 °C increase in thermal stability of Gln143Asn MnSOD relative to the wild-type enzyme.

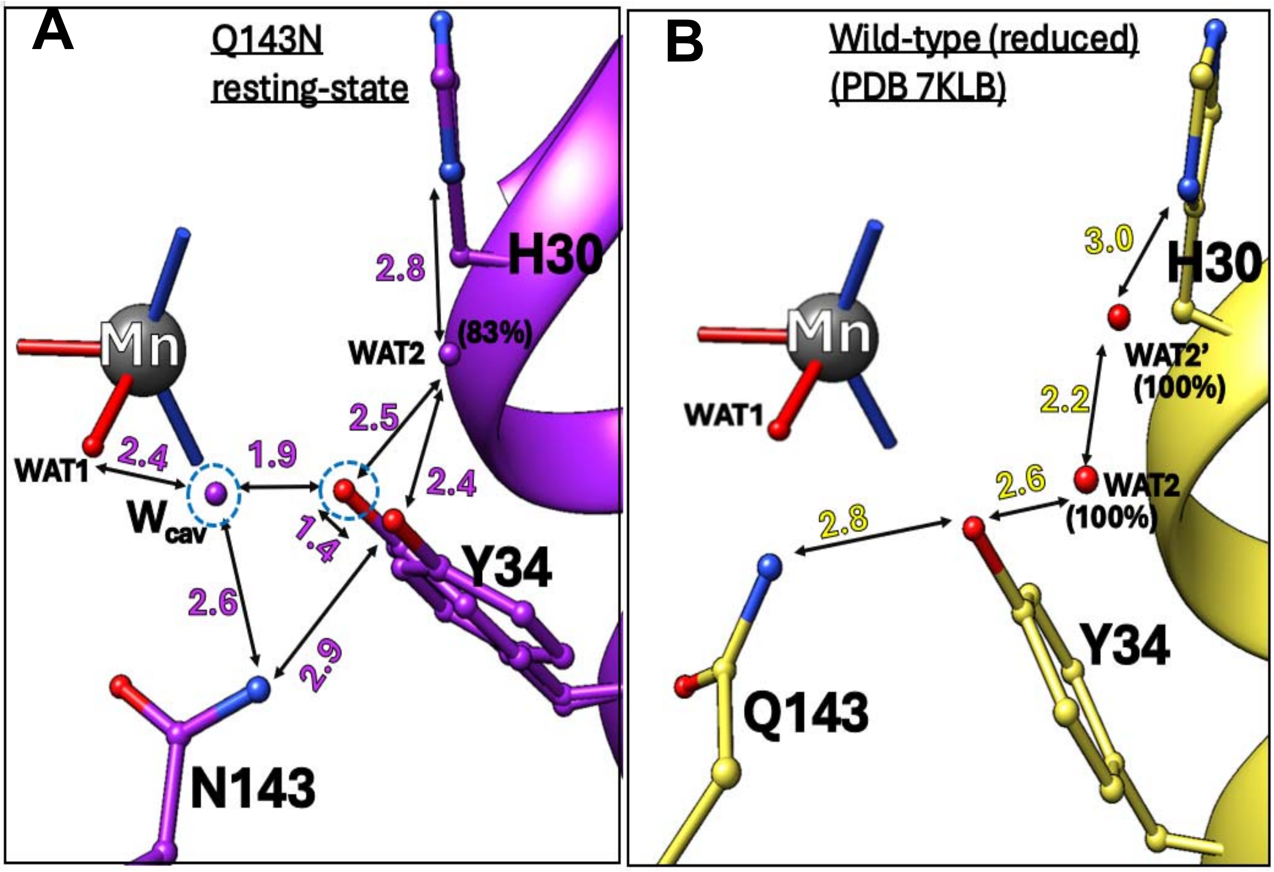

Figure S4 Gln143Asn MnSOD supports a partially hexavalent Mn coordination environment. **(A)** and **(B)** show the resting-state and H_2O_2 -treated active sites of Gln143Asn MnSOD respectively. display significant difference electron densities consistent with the presence of partially occupied sixth ligand (named L_{six}), likely a hydroxide ion (OH^-), opposite Asp159, observed at 23% occupancy in the resting state, and 17% following H_2O_2 exposure. This sixth ligand has previously been reported, only at full occupancy in chain A of the reduced human wild-type Mn^{2+}SOD (PDB 7KKW). Difference densities for the modelled W_{cav} and L_{six} in both structures of the Gln143Asn are represented as mesh, coloured according to their individual contour levels. Presence of this partial sixth coordination partner induces a partial distortion of the Mn^{2+} geometry toward octahedral, diverging from the canonical trigonal bipyramidal configuration characteristic of the penta-coordinate active site

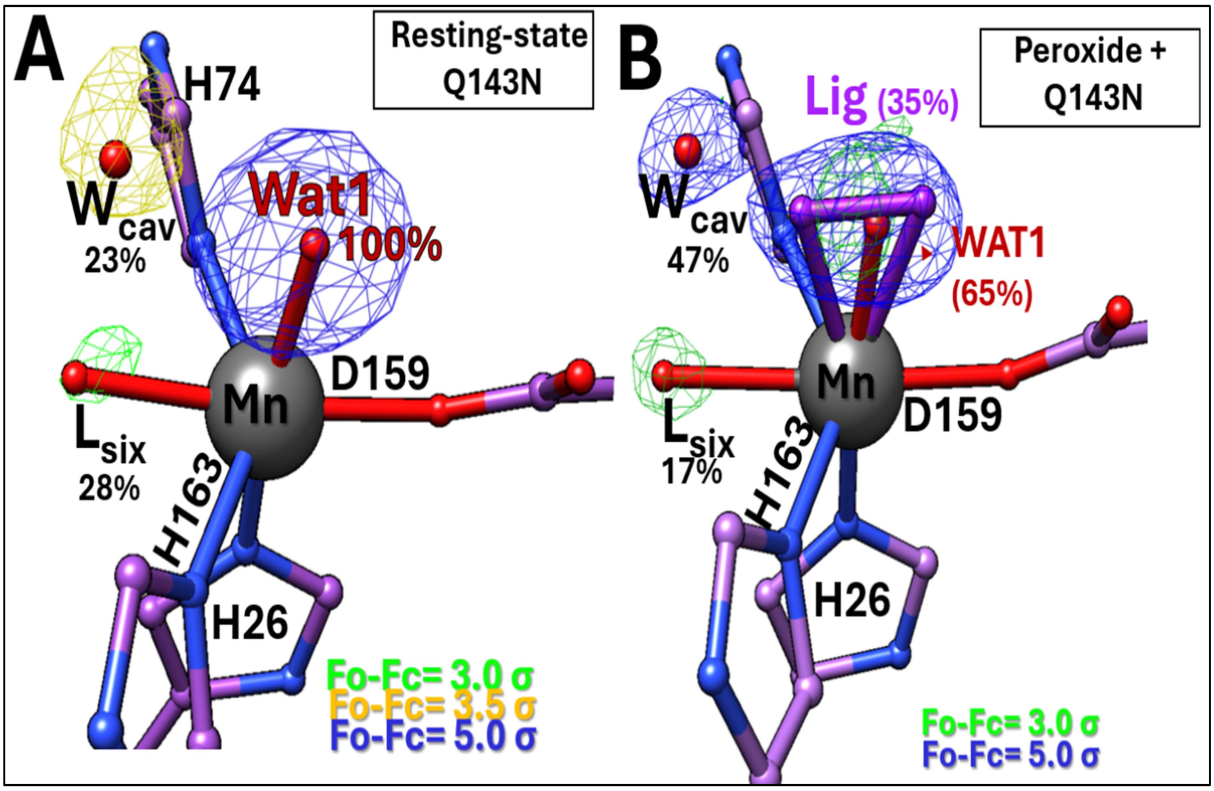

Supplement: Supplementary file 1 [file f-81-00467-sup1.pdf]
